# Application of Two-Eyed Seeing in Adolescent Mental Health to Bridge Design Thinking and Indigenous Collective Storytelling

**DOI:** 10.3390/ijerph192214972

**Published:** 2022-11-14

**Authors:** Johanna Sam, Chris G. Richardson, Leanne M. Currie

**Affiliations:** 1 Department of Educational and Counselling Psychology, University of British Columbia, Vancouver, BC V6T 1Z4, Canada; 2 School of Population and Public Health, University of British Columbia, Vancouver, BC V6T 1Z3, Canada; 3 School of Nursing, University of British Columbia, Vancouver, BC V6T 2B5, Canada

**Keywords:** mental health, adolescence, Indigenous methodologies, information and communication technology

## Abstract

Background: eMental health apps are increasingly being considered for use in health care with growing recognition of the importance of considering end-user preferences in their design and implementation. The key to the success of using apps with Indigenous youth is tailoring the design and content to include Indigenous perspectives. In this study we used a Two-Eyed Seeing perspective to integrate Indigenous and human computer interaction methodologies to identify end-user preferences for a tablet-based mental health screening app used in a primary care clinic serving Indigenous youth. Objective: The research objectives used a Two-Eyed Seeing approach to (i) collectively create stories about Indigenous youth lived experiences accessing integrated primary care for their mental health concerns; and (ii) engage Indigenous youth in Design Circles to determine their usability preferences for digital mental health screening tools. Method: Eight adolescents (n = 4 young women; n = 3 young men; and n = 1 Two Spirit) between 20 to 24 years old who self-identified as Indigenous participated. Indigenous youth joined Design Circles to co-create a story about accessing mental health care and their needs and preferences for an eMental Health app. Results: Findings highlighted the importance of collective Indigenous storytelling about accessing integrated primary care for mental health needs. Participants created three persona stories about their challenges accessing mental health care and the role of social support. Participants sorted their usability design preferences for an eMental Health app to be inclusive of Indigenous knowledges. Conclusions: A Two-Eyed Seeing perspective was useful to incorporate a design thinking approach as collective storytelling among Indigenous youth. This research may inform and shape the design of eMental health apps used in health clinics to better engage Indigenous youth.

## 1. Introduction

eMental health apps (i.e., software applications designed for use on mobile devices such as smartphones) are increasingly being considered for use in health care settings [1] and the incorporation of end-user perspectives has been shown to enhance the design of such apps [2]. In the mental health field, scholars have advocated for a “public mental health” approach that takes a population perspective to target the treatment of mental health disorders and promotion of wellness [3]. eMental health screening tools that can be used to assess the mental health and wellbeing of youth represent an important component of prevention and early intervention efforts that are based on this public health approach [3]. A major challenge in the development and implementation of mental health screening is the provision of culturally safe care to Indigenous people. In this study, we sought to bridge Indigenous research methodologies with user-centered design methods to identify ways to engage Indigenous end-users in the design process of an eMental health screening app in a culturally safe manner. The eMental health app in this study was an iPad-based mental health screening tool to identify youth as having one or more mental health problems, including internalizing or externalizing mental disorders, substance use disorders, or violence problems [4]. Toward this goal we explored the preferences of Indigenous youth who would be the end-users of the eMental health screening app designed to be completed prior to a visit in an integrated primary care office. 

eMental health screening apps can capture areas of concern noted by youth and generate conversation with clinicians about mental health issues. Effective and early eMental health screening supports a public health approach, which often leads to better health outcomes and recovery [5]. Our goal was not to design/re-design the eMental health screening app, but rather explore the potential of integrating methods from human–computer interaction research, specifically Design Thinking, with Indigenous Storytelling as a potential methodology for eMental health screening software design with Indigenous youth. In order to bridge these two apparently disparate perspectives, the current study used a Two-Eyed Seeing approach to explore the collective experiences of Indigenous youth accessing integrated primary care for mental health concerns as well as their preferences for digital mental health screening tools. Briefly, Two-Eyed Seeing refers to learning to see the strengths of Western methodological approaches from one eye and the strengths of Indigenous knowledges from the other eye, and to use both eyes together for the benefit of all [6]. The linkages between the three concepts (Two-Eyed Seeing, Design Thinking, and Indigenous Storytelling) will be further described below.

Mental health disorders and substance misuse represent leading causes of diseases for adolescents and young adults [7]. While Indigenous young people are resilient, they often experience a higher burden of mental health illness and life stressors, such as family obligations and lack of cultural supports [8,9,10]. These disparities relate to historical and present manifestations of colonialism, particularly intergenerational trauma of residential schools and Indian hospitals, forced displacement to reservations, and mental health service gaps [11,12,13]. This study uses the term Indigenous to describe the First Peoples of Canada, particularly First Nations, Métis, and Inuit. There is widespread recognition that a better understanding of Indigenous perspectives is needed to create innovative, culturally revitalizing mental health screening resources and supports [14,15]. 

The lack of culturally safe digital mental health screening in Indigenous adolescent populations, who are commonly at highest risk for mental health problems, is problematic. In this study, the eMental health screening tool and culturally revitalizing Indigenous methods are combined to address a gap in a public health approach of early detection and prevention. The purpose of this study was to use a Two-Eyed Seeing approach to examine the preferences of Indigenous youth toward the eMental Health screening app. There were two specific research objectives when examining the preferences of Indigenous youth using an eMental health screening tool. The first study objective was to collectively create stories about Indigenous youth lived experiences accessing integrated primary care for their mental health concerns. The second study objective was to engage Indigenous youth in Design Circles to determine their usability preferences for eMental health screening tools. Indigenous youth ages 15 to 24 were invited to participate in this research because this age represents a developmental period when mental health problems emerge [16]. The present study involved interweaving Western methods, Design Thinking, with Indigenous methods, specifically traditional Indigenous Storytelling and oral traditions. The present study also draws attention to lived experiences of Indigenous youth when accessing integrated primary care for mental health needs in addition to their preference of eMental health screening tools. 

Adhering to Indigenous protocols, we first locate ourselves in relation to the current study [17]. We are a collective of scholars who work in public health in western Canada. As Indigenous and allied scholars, we came together with the intention to understand how we might Indigenize an eMental health screening app for adolescents accessing primary health care delivered in an integrated clinic setting. Johanna is a proud citizen of Tŝilhqot’in Nation in north-central British Columbia. Johanna’s research takes a strength-based approach to bring Indigenous ways of knowing into education and mental health to create safe digital spaces for youth. Chris is of Settler heritage and is a step-parent of three Indigenous daughters of the Selkirk First Nation. His research focuses on early identification and intervention efforts to support youth with mental health and substance use challenges. Leanne is of Métis, Cree and Settler heritage and a member of the Métis Nation of British Columbia. Leanne’s research is in the field of nursing and health informatics with a goal of creating joyful spaces for design to happen and culturally safe software.

### 1.1. Indigenous Youth Mental Health

The impact of colonialism as a social determinant of health has been shown to be devasting on Indigenous youth populations [11,13]. To understand mental health disparities among Indigenous adolescents, the harms from colonialism that furthered the goal of assimilation by forcible displacement from land, outlawing Indigenous traditions and governance structures, spread of disease (e.g., smallpox), assimilative policies, and cultural genocide [18] must be understood. In Canada, the Indian Residential School system was in place from the 1830s until 1996 [19]. The Canadian Indian Act, instituted in 1876 and still in force with amendments, legally enforced the removal of Indigenous children and adolescents to attend Indian Residential Schools, which forbade Indigenous children to speak their language and practice traditions [19]. Many Indian Residential School survivors experienced sexual, physical, and emotional abuse while attending Indian Residential Schools [19]. Mental health disparities among Indigenous adolescents can be understood as rooted in the legacy of residential schools and ongoing colonialism. Recent research indicates that intergenerational trauma associated with Indian Residential Schools continues to negatively impact Indigenous youth today [20]. In particular, Indigenous adolescents who had a grandparent or both a parent and grandparent attend an Indian Residential School were more likely to be involved in the child welfare system [20]. 

In the report A Portrait of Youth in Canada, Indigenous youth were noted to be less likely to indicate excellent or good mental health in comparison to Indigenous adults and older adults [16]. A community-based longitudinal study with nearly 700 Indigenous adolescents living on a reservation in either Canada or USA found that 77% had a cumulative lifetime prevalence for any mental health disorder and 56% had a prevalence for comorbid mental health disorders [21]. In Canada, nearly 19% of Indigenous youth had been diagnosed with a mood disorder and 24% had been diagnosed with an anxiety disorder [16]. In regard to comorbidity, Indigenous adolescents commonly experienced substance use disorder along with generalized anxiety or mood disorders [21]. Further, in Canada, young Indigenous women were more likely to report mental health issues compared to young Indigenous men [16]. Generally, comorbid mental health disorders, such as substance use and internalizing problems, were higher among young Indigenous women on a reservation in comparison to young Indigenous men living on a reservation in Canada or USA [21].

Despite negative mental health outcomes, researchers and clinicians know that Indigenous youth often attempt to seek health care support [14]. A national Canadian report indicates that Indigenous people living in an urban centre have better access to a range of mental health care services in comparison to those living in a rural and remote community [14]. Furthermore, there is growing recognition of the benefits of early detection, intervention and health promotion in adolescence [5]. Despite access to integrated primary care services, many Indigenous people in an urban centre report barriers to care including racism, long wait lists, and culturally unsafe care [14]. Moreover, there are relatively few culturally safe, effective, and evidence based eMental health resources to engage Indigenous youth [22]. Nevertheless, widespread availability of mobile devices and Wi-Fi connectivity provides an opportunity to remove barriers to mental health care access within underserved communities and marginalised youth [23]. Yet, there is a dearth of research that has investigated the preferences of young Indigenous people in eMental health screening tools. 

### 1.2. Two-Eyed Seeing, Indigenous Methodologies and Design Thinking

The present study is rooted in Indigenous methodologies and knowledges to further reconciliation in adolescent mental health care. To this end, the current research applied an Indigenous conceptual framework known as Two-Eyed Seeing to explore Indigenous youth lived experiences on how they access integrated primary care for their mental health needs. Two-Eyed Seeing was used as the guiding principle to bridge Design Thinking with Indigenous oral traditions of storytelling to incorporate a more holistic understanding of Indigenous youth lived experiences. In this work, we suggest that a Two-Eyed Seeing framework is a novel transcultural approach that can combine Indigenous and Western methodologies in adolescent mental health care to explore access to integrated primary care for their mental health needs and preferences for eMental health screening tools. 

A systematic review on technology-based youth mental health interventions discusses the importance of end-user involvement in the development and evaluation of digital mental health screening resources [2]. A collaborative process is undertaken in Design Thinking to design, review, and modify eMental health screening resources [2,24]. Design Thinking can be seen as a participatory approach that is an iterative process, which involves the end-users in the co-creation and evaluation of digital resources, including eMental health screening apps [24]. Despite the importance of end-user involvement, there remains a lack of Indigenous knowledges and methods undertaken in participatory Design Thinking approaches. 

Indigenous storytelling promotes the balance of intellectual, physical, emotional, and spiritual traditions for a healthy lifestyle. In Indigenous oral traditions, “people keep the spirit of a story alive by telling it to others and by interacting with the story” [25]. Using the Two-Eyed Seeing approach we have coined the term ‘Design Circles’ to capture the integration of Design Thinking and Indigenous Storytelling. The term *circle* is use symbolically here because Indigenous Storytelling as a methodology often incorporates the physical act of sitting in a circle where everyone can see all others who are listening to the storyteller, thus all are engaging with the story, the storyteller, and all others in the circle. Indigenous people come from an oral tradition of storytelling, “and as storytellers, [they] have a responsibility to be honest, to transmit [their] understanding of the world to other people” [26]. Storytelling provides an opportunity for knowledge sharing that gives strength between the storyteller and listener, and was a way to teach younger generations [26]. Listeners often take cultural values of respect, responsibility, reciprocity, and relevancy to learn how to make meaning from stories [26,27]. Stories are interconnected with the world, families, communities, animals, and culture [26]. Oral stories are shared relationally, which may “hold personal narratives of place, happenings, and experiences” [17]. The desire for a deeper understanding of collective storytelling connected to Design Thinking prompted the strengths-based investigation in the present research. 

We conceptualise Design Circles in the present study to allow for culturally revitalizing expressions of Indigenous storied traditions through collective narrations. The term Design Circles is also intended to respect the voices and agency of Indigenous adolescents, especially as storytelling can support and uphold Indigenous knowledges and oral traditions. Lopez-Carmen and colleagues [28] found in their international review of eleven studies, that the involvement of community workers, participatory frameworks, cultural perspectives, as well as formal and informal discussions are key components to improve Indigenous children and adolescents’ wellness. Joan Bottorff and colleagues’ [29] research on designing tailored health messages indicates the importance of including the views of Indigenous youth. While Indigenous youth perspectives were similar to those of their non-Indigenous peers, the Botorff et al. findings suggest a need for cultural symbols as starting point for Indigenous youth to engage with the health messages [29].

Research focused on how to interweave Indigenous methods, knowledges, and oral traditions with Western methods, specifically Design Thinking, to create digital mental health screening resources are almost non-existent. The idea of Indigenizing a mobile mental health screening app in an integrated primary health clinic was not about replacing Eurocentric approaches to health with Indigenous knowledges. Instead, it was about weaving Indigenous youth experiences and knowledges to modify the design of the eMental health screening app in integrated primary care. To respectfully build relationships with Indigenous peoples and communities, it is essential to use an Indigenous methodology that centres several aspects: (i) holistic epistemology; (ii) story, purpose, the experiential; (iii) Indigenous ways of gaining knowledge; and (iv) consideration of colonial histories [17]. We draw on elements from an Indigenous research method guided mostly by a Nêhiýaw worldview [17]. Specifically, the present study draws on an Indigenous research framework with the Nêhiýaw worldview including: Indigenizing ethics; research preparations involved knowledge gathering procedures that weave together cultural protocols and Western research design; making meaning of stories gathered; and giving back [17]. Thus, story and Indigenous knowledges are situated within relationships, which honours voices in a variety of methods (e.g., conversations in Circles or focus groups [17]). Further, a Design Circle provides a way for Indigenous people to share their stories collectively that is not constrained by a structured interview [17].

Given the need to create responsive and holistic care options, the current study aimed to address this gap by exploring Indigenous young people’s usability perspectives to guide the design, layout, symbols, and formatting of an eMental health psychosocial screening tool, TickiT [30] that was installed on tablet computers (e.g., iPad) at Providence Healthcare’s Inner City Youth Programs’ clinics. The present study used a Two-Eyed Seeing approach to focus on the following research question: What are the collective experiences of Indigenous youth accessing integrated primary care for their mental health and what are their preferences for digital mental health tools?

## 2. Methods

### 2.1. Study Setting

The present study was carried out within the Providence Health Care’s Inner City Youth Program, which had a pre-existing relationship with the Urban Native Youth Association (UNYA). The Inner City Youth Program established an integrated primary care clinic on the UNYA premises. It was common for primary care and mental health care services to be separate, but in an effort to provide holistic wellness to adolescents and to provide better mental health care, integrated primary care combines the two to become one service called integrated primary care. The community-based integrated primary care delivery model provided Indigenous youth an opportunity to access a wide range of mental health and substance use health care while remaining in or close to their home communities. The Inner City Youth Program at UNYA served Indigenous adolescents residing in an urban centre located in Southern British Columbia, Canada. The research team worked in collaboration with UNYA staff to coordinate the research. 

### 2.2. Recruitment and Participant Characteristics

We shared invitations to participate in two Design Circles in collaboration with UNYA staff to distribute study brochures, on-site posters, and announcements on an email listserv and social media (e.g., Facebook). Adolescents were eligible to participate in the Design Circles if they self-identified as Indigenous (e.g., First Nations, Métis, or Inuit), were between 15 to 24 years old, and were available to attend two Design Circle sessions in person. All participants attended both Design Circles.

At the end of the first Design Circle, participants completed a short questionnaire that consisted of two parts: (i) demographic information and (ii) technology knowledge and use. Socio-demographic information included: (a) month and year of birth; (b) highest education; (c) sex and gender; (d) Indigenous identity (First Nations, Inuit, or Metis); and (e) living situation (e.g., asked which persons does they live with). Their technology knowledge was gathered using three measures. First, participants were asked about their use of mobile technology, and different aspects of their Internet use, including an estimate of the amount of time spent online. Next, participants completed the eHEALS tool as a self-report on their eHealth literacy to estimate their online health skills to inform their wellness decision making and health planning. The eHealth Literacy Scales (eHEALS) consists of 8-items. Items are scored on a 5-point Likert scale ranging from strongly disagree to strongly agree. The eHEALS has been shown to have strong internal consistency (α = 0.88) [31]. In this sample, the Cronbach’s alpha for eHEALS was α = 0.72. A mean score was calculated for each item of the eHEALS scale. Higher scores represent higher self-perceived eHealth literacy skills. Finally, participants were asked about their perceptions of trustworthiness of health information on the internet via the following question “when using the internet to find health information, how do you know that you can trust the information that you found?”

Two Design Circle sessions were attended by a total of eight individuals. Each session lasted approximately two-hours. Average participant age was 22 years (range = 20–24 years old). Participants were young women (n = 4), young men (n = 3) and Two Spirit (n = 1). For many Indigenous people, Two Spirit is the preferred term for individuals who do not identify as heterosexual [32]. The majority of participants (88%) had at least graduated high school and 38% of participants had an education beyond the high-school level. A total of four participants had lived in an urban centre for more than ten years, and four participants had a regular family physician. The majority of participants (75%) self-identified as status First Nations. Most participants (75%) lived independently without their caregiver. Table 1 provides an overview of participant sociodemographic characteristics. 

Participants most often use a mobile phone (n = 3) to access the internet. Five of the 8 participants (60%) reported accessing the internet seven days a week. Nearly 70% of participants owned their own device. Approximately 60% of participants indicated that it was not at all or somewhat important to be able to access the internet (please see supplemental Table S1). 

Mean scores of the eHEALS are shown in Table 2. Results of these mean scores indicated that participants agreed that they knew where (M = 3.88, SD = 0.35) and how (M = 3.88, SD = 0.64) to find helpful online health resources in regard to their own concerns. Yet, participants self-reported that they felt less confident in using the online health resources to make a decision about their own health matters (M = 2.88, SD = 1.13). 

Participants indicated several strategies that they use when determining the trustworthiness of online health information (shown in Table 3). A majority of participants (63%) verify trustworthiness of online health information if the website belonged to a health organization, hospital or government. Fifty percent of participants reported that they perceived online health information as trustworthy if they recognized the group or person who created it. None of the participants reported that they determine trustworthiness of the online health information by relating to the people who developed the website.

### 2.3. Ethics

We obtained informed consent from all participants included in the study. There are some unique cultural considerations that may arise from consent procedures. Specifically, Indigenous people are traditionally oral societies and written consent may be seen as not respecting Indigenous approaches to research initiatives. Oral consent was an appropriate alternative to obtaining written consent. As per CIHR Guidelines for Health Research Involving Aboriginal People, a research team member documented the date, time and place in which the oral consent of a participant was received. To assure participants’ confidentiality and anonymity, we have removed identifying information from collective storytelling. This study received ethical approval from the University of British Columbia’s behavioural ethics committee harmonized board of Providence Health Care Research Ethics on June 2016 (certificate number: H15-02180). Each participant received a $25 gift card as well as had their named entered to win one $500 gift. 

### 2.4. Knowledge Gathering Procedures

We conducted both Design Circles within a two-day period. Four researchers, two graduate students and two faculty members, facilitated the Design Circles in August 2016. We scheduled the Design Circles sessions to last two to three hours (Table 4). The Design Circles were semi-structured to facilitate collective discussion and storytelling. 

The session began with an ice-breaker task in which participants asked each other questions to get to know one another. Following this, we introduced the eMental health screening app used by integrated primary clinics and asked them to think how other Indigenous youth across the province may feel using the app prior to an integrated primary care professional (e.g., medical doctor or nurse practitioner) appointment. Then, we separated participants into three smaller groups. Each group was provided with a large (3 foot by 4 foot) sheet of paper to collectively create a story of an Indigenous youth who might use the eMental health app in the clinic waiting room. Participants worked collectively to complete two design thinking activities called empathy-mapping and persona creation. Each group invented fictitious demographic characteristics and a false name for their persona story. As they developed their persona story, they ascribed thoughts, feelings and behaviours to each personal health journey narrative. Empathy mapping is a visual collective method to develop a personal narrative in four quadrants to describe what an Indigenous youth might “think and feel”, “see”, “say and do”, and “hear” [33]. Empathy maps served to centre the discussion on the ‘future end-users’ to discuss Indigenous youth wellness without self-disclosing personal circumstances. Past research found that persona development increased openness from participants when discussing experiences which may evoke stigma [34]. Each group of participants told one story about Indigenous youth experiences when accessing integrated primary care for their mental health needs. 

Participants were given tablets to interact with the eMental health app and review the waiting room questionnaire. It took participants approximately 5 to 10 min to review all the screens and questions on the eMental health app together. In small groups, participants either worked independently on eMental health app solutions that address the created storytelling persona stories. The participants were asked to use the eMental health app based on their persona to evaluate usability, attitudes towards certain interface elements (e.g., thumbs up/down answer options, types of questions, health quotes on screen, overall appearance, colors, and layout). Participants were provided with supplies (e.g., pens, pencils, felt markers) to co-develop a new interface design and layout to better fit their group’s Indigenous youth persona needs.

The second Design Circle was opened by sharing the British Columbia First Nation Health Authority’s perspective on wellness with participants. Participants viewed a news story on YouTube about Indigenous people’s experience with racism in Canadian healthcare. The facilitators led a debrief after the video by asking participants to consider Indigenous values and beliefs, especially holistic wellness (e.g., teachings, history, culture, ceremony, family and community). The last activity of the Design Circle ended with sorting design usability preferences activity. Each participant was given a stack of post-it notes and were asked to write a single concept on each post-it note. Concepts were related to Indigenous ways of knowing to guide usability and design of the eMental health app. The groups were asked to sort the post-it notes they created by grouping related concepts into themes or key concepts. 

### 2.5. Data Analysis 

One of the bridges between Indigenous and Western methods is the data analysis of persona stories. Using the Two-Eyed Seeing approach means that data analysis is contextualized as Indigenous storytelling as a form of personal narrative to make meaning of participants’ lived experiences. We transcribed the collective storytelling into persona stories through a process of design thinking in a game-storming format [33]. This process allowed participants to engage in collective storytelling of a health care journey to discuss potential challenges faced by Indigenous youth. All visual images created during design thinking as collective storytelling were photographed. Following the Design Circle sessions, the authors debriefed about design thinking approach and how it relates to collective Indigenous storytelling. Analysis was carried out by analysing personas derived from the collective storytelling process. The three personas that were created by participants each had individual characteristics that highlight the collective wellness needs and strengths of Indigenous youth. The personas share a unique story about a young Indigenous person’s health journey and their interconnectedness to others, their culture, and community. We then input data into Microsoft Excel to support the organization of analysis of demographic questionnaire responses. The descriptive statistics were calculated (i.e., M, SD) using IMP SPSS Version 25 (IBM, Corp, 2017). 

## 3. Results

Collective storytelling engaged participants to collaboratively create a personal mental health journey narrative experienced by an Indigenous youth. The Design Circles produced lively collective storytelling among Indigenous youth. Indigenous multimodal meaning making draw on elements of storytelling, including images and drawings, as a valid representation in which Indigenous youth engages. The qualitative data for the current study were based on text entries and photographs of responses. 

### 3.1. Overview of Collective Storytelling

**Figure 1:**
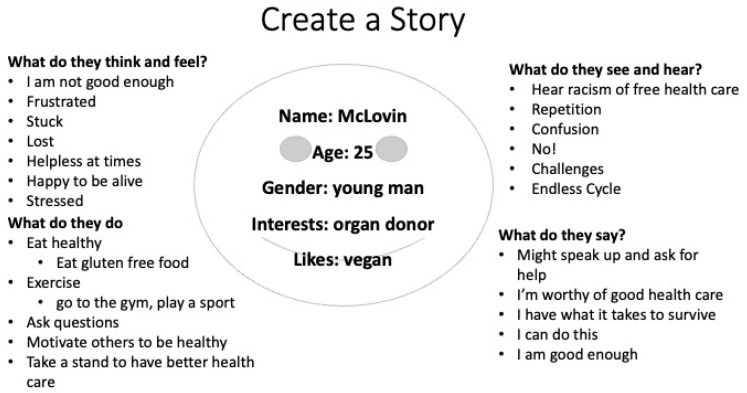
Empathy map for persona story, McLovin.

The three persona stories, Donald Duck, McLovin, and Sage, were developed collectively by participants in small groups. The personas highlighted the barriers to access mental health care. The persona stories evolved to show evidence of systemic racism in health care. Each persona story describes how Indigenous youth strive to stay healthy and thrive. Persona stories expressed how they seek help from others. Figure 1 is a representation of the empathy map created by the participants. The persona stories are shared below as collective storytelling narratives. 

#### 3.1.1. First Persona Story

Donald Duck is a 17-year-old male. He’s attending high-school. He likes PE class. His least favourite classes are English and Math. Donald enjoys being physically active especially, running, hiking, and contemporary and traditional Indigenous dance. He likes listening to music. When Donald gets sick, he waits until it gets really bad before he gets help. He feels it is hard to get help from doctors, so if it’s not a big enough problem he will not go into a clinic. Donald will wait for the sickness to get worse, for example, a cold, leads to the flu, and turns into an infection. Donald may miss school from being sick. While at home, he will stay connected with friends and family on social media, like Facebook. He feels happy to be online, since he has something to do. He gets advice on Facebook from his friends about what is making him sick. Donald may search Google for his symptoms. He finally decides to go to the walk-in clinic. He gets medication, such as antibiotics. Yet, he does not get better. He goes to the hospital because the doctors are better. The hospital is a safe place that has food and a bed. He feels nervous, yet happy at the hospital. He is happy to get help, but nervous about what will happen. He is also happy to be at the hospital because he does not want to get his family members sick. Donald is able to get all the help he needs at the hospital because it has everything from tests to medication. He does not have to go to the pharmacy to get his medication when leaving the hospital. Donald gets better. He is able to go back to the community to dance. He enjoys dancing because he can teach younger dancers. He also serves Elders first at community. He stays connected. 

#### 3.1.2. Second Persona Story

McLovin is a 25-year-old male. McLovin is an organ donor. He is in college studying. He exercises by playing disc golf and beach volleyball. He walks to a liquor convenience store. Sometimes he’s happy, but other times he’s sad. There is racism around him. He hears others saying “why do you get free healthcare when others don’t?” He feels judged because some people assume that all Natives are the same. He tries to eat healthy food to maintain his health. He might speak up to ask for help. He says, “I have what it takes to survive”.

#### 3.1.3. Third Persona Story

Sage is an 18-year-old young woman who has grown up on her reservation with her First Nations community. She likes to be involved in her culture. She has a cultural identity. However, she is scared to go to the clinic and doctors because her family has told her scary stories. Sage has been having sex. She is worried that she has a sexually transmitted infection (STI), but does not want to go to the doctors. She is scared about what will happen and what they will do at the clinic. 

### 3.2. Overview of Design Usability Preferences

**Figure 2:**
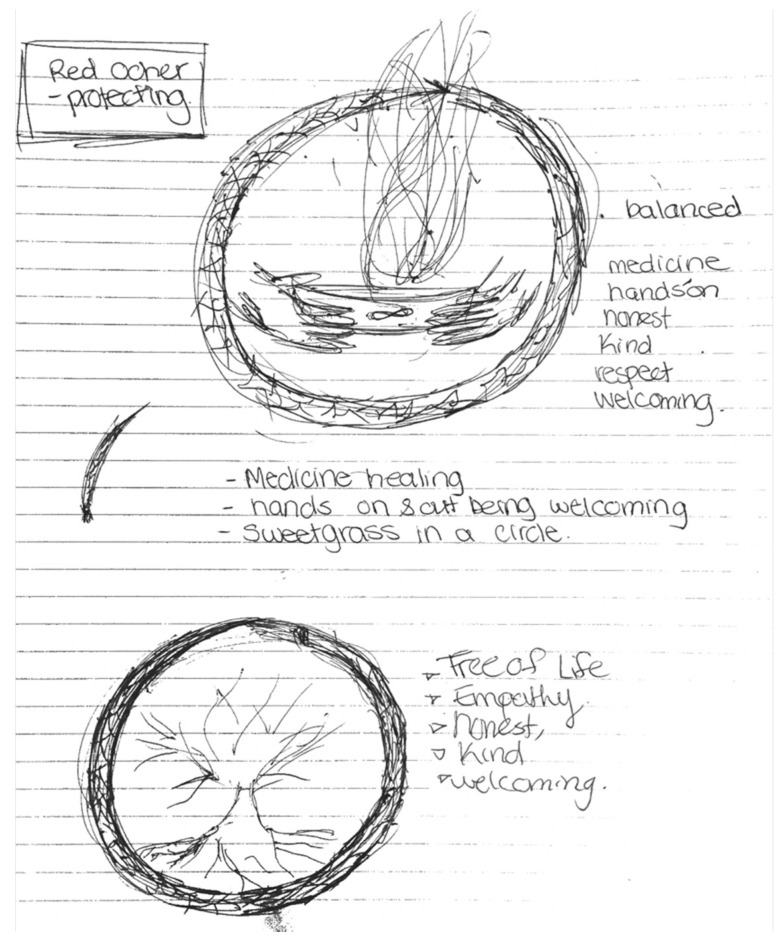
An Indigenous Interface Design Usability Preferences, The Sacred Tree teachings.

Participants generated ideas for a new interface design and layout grounded in Indigenous knowledges and cultural wellness. Usability was investigated through Indigenous storytelling and oral traditions about perceived cultural safety and inclusive of Indigenous knowledges on wellness. Figure 2 illustrates that in order to live well and have a balanced life, traditional medicines from an oral story of the Sacred Tree must be included in one’s life [35]. The illustration shows Sacred Tree teachings in several ways, including sweetgrass in a circle, empathy, honesty, and respect. The Sacred Tree teachings are embedded in the imagery and words chosen [35]. Sweetgrass is use in traditional healing ceremonies to symbolically repel negative energies and protect one’s living space, environment and self. Sweetgrass is also used for ‘smudging’ where the grass is burned and the person is encircled in the smoke that is produced. Smudging ceremonies are often part of spiritual experiences for many Indigenous groups, and are seen as a way to provide a sacred and spiritual healing experience [35]. Red ochre is also a believed to have protective properties and has been associated with connection with a tribe as well as the ability to protect against evil. Indeed, recent reconnection to traditional spiritual practices has identified use of red ochre for face painting as a way of “reclaiming culture” [36].

The tree of life is a powerful source of Indigenous health and wellness. The roots of the tree signify connection with Mother Earth, and the branches reaching to the sky indicate the linkage to the Creator. The teachings of the Sacred Tree indicate that “the life of the tree is the life of the people” [35]. Thus, through the story of the Sacred Tree, the participants were able to provide a visual illustration to signify healing and wellness. 

**Figure 3:**
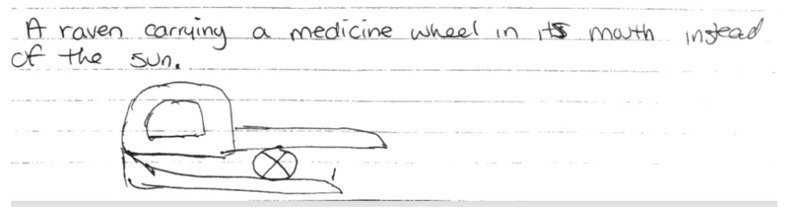
An Indigenous Interface Design Usability Preference, The Raven and the Sun.

A second illustration (Figure 3) developed by participants focused on Indigenous oral traditions, specifically a creation story. The image of the Raven and the Sun depicts a creation story about how humans received the necessities of life [37]. The participants used an image of the Medicine Wheel instead of the sun to centre on balanced wellness as a necessity to life. 

**Figure 4:**
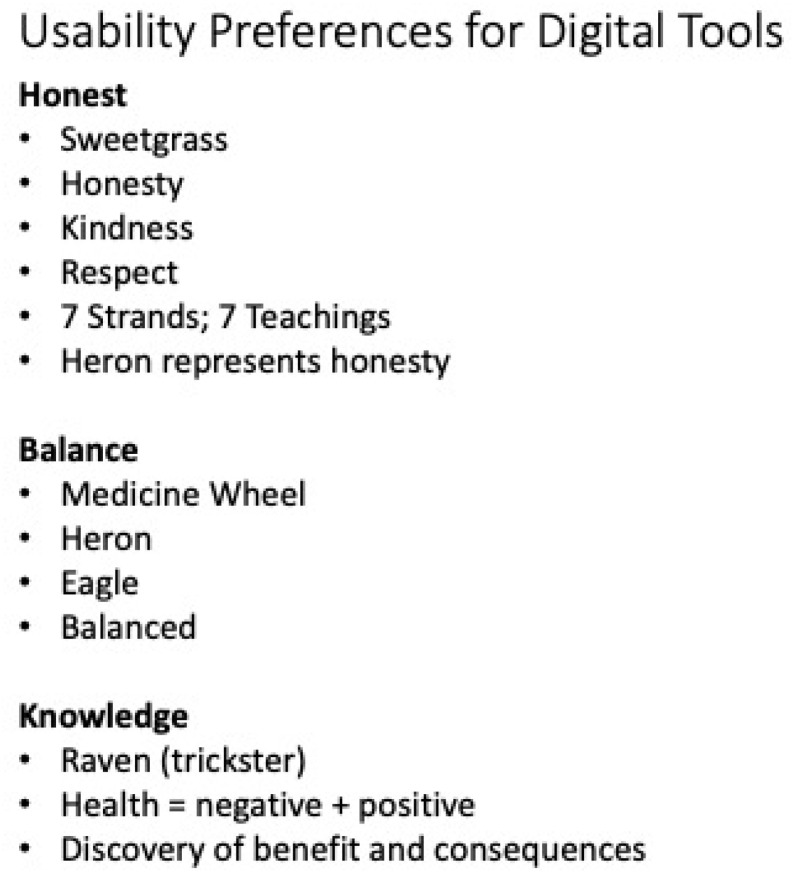
An Indigenous Interface Design Usability Preference, Words Chosen in Cultural Meanings of Imagery.

Figure 4 shows some of the cultural meanings behind the imagery used in their design usability preferences for a mobile app. The illustrations were brought together as a collective storytelling approach when describing the cultural meaning behind the images. There was a focus on honesty, which the circle of sweetgrass represented. The sweetgrass has seven strands to represent each of the seven sacred teachings braided into cultural healing practices. The importance of a balanced lifestyle as essential for wellness was shown in the Medicine Wheel. There was a recognition of knowledge, which was depicted in the Raven who is often a Trickster in oral traditional stories by teaching others about negative and positive lifestyle choices. All of the imagery used were grounded in oral stories and cultural traditions. 

## 4. Discussion

Advancing eMental health screening tools represents an important element of public mental health, and it is likely that there will be a growing demand for culturally safe eMental health apps. This research applied a Two-Eyed Seeing approach to explore the intersection of a Western method of Design Thinking and Indigenous method of Storytelling towards creating a collective story about young Indigenous people experience accessing integrated primary care for their mental health needs in addition to their usability preferences for eMental health apps. To the knowledge of researchers, the present study is the first use a Two-Eyed Seeing approach to weave together Design Thinking and Indigenous collective storytelling to understand Indigenous youth experience when accessing integrated primary care for mental health care and usability preferences of digital mental health screening tools. The impact of this study using Design Circles to engage Indigenous youth about their collective experiences when accessing integrated primary care for their mental health needs is indictive of their usability preferences as future end-users to be more relevant in the creation of digital mental health screening tools [38]. Two-Eyed Seeing offered an opportunity to interweave participatory design thinking to Indigenous Storytelling methodologies to give Indigenous youth voice to promote their sense of agency when identifying solutions for themselves and Indigenous communities. Researchers suggest that effective digital mental health screening resources should be developed in collaboration with youth, especially from underserved communities [23]. 

Narrative study findings from persona stories each had unique characteristics that highlight the individuality and collective experiences of Indigenous youth who are accessing integrated primary care for their mental health needs. For example, the first persona story described how Indigenous youth may delay seeking health care immediately, only going to a hospital when severity of health concern increases. The first persona story revealed the important aspects of Indigenous views of wellness, including culture, Elders, family, traditions, intergeneration, and community. Particularly, the first persona story identified how Indigenous youth may seek health advice online, either by searching for information on websites or social media networking. The second persona story illustrated racist comments heard by Indigenous youth in health care. The second persona story recounted eating healthy and importance of personal resiliency. The second persona story reported having personal strength speaking up to ask for help from others. The third persona sought help from family members. The third persona story reveals staying involved in culture and community. The third persona story describes being worried about what will happen at a clinic. 

One of the main findings that this work highlights is the importance of the application Two-Eyed Seeing approach in Design Thinking and Indigenous storytelling to create culturally revitalizing digital mental health screening tools. The results of the current study are supported by Ellis and colleagues’ 2020 [39] meta-analytic study findings that showed culturally adapted eMental health interventions contributed to positive health outcomes among racial and ethnic minority communities. In particular, their meta-analysis highlighted that culturally safe eMental health interventions lessened mental illness symptoms and improved quality of life [39]. We found that Indigenous storytelling underpinned all aspects of design usability preferences for eMental health screening tools. First, a collective Indigenous Storytelling approach in Design Thinking can be considered a participatory process for co-creating persona stories to identify integrated primary care access barriers. Then, the usability design preferences demonstrate how aspects of culture can be embedded within a digital health tool and how cultural knowledges can be respected on digital mental health resources. Pivotal strategies for embedding culture included the recommendation to incorporate oral stories and traditional healing practices into digital health tools. Culture may be strengthened by ensuring oral storytelling and views of balanced wellness are incorporated into digital mental health resources. Similarly, Lopez-Carmen et al. [28] reported cultural sensitivity as a key factor to improve the mental health of Indigenous youth. They state that effective early mental health interventions for Indigenous youth requires an understanding of Indigenous knowledges on wellness, respectful communication, and cultural values. 

Indigenous youth commonly face health care access barriers and discriminatory systems. The present study identified underlying reasons that may delay young Indigenous people from seeking integrated primary care for their mental health needs. Discrimination by others including health care professionals was reported in the persona stories. Racism and shame, particularly about mental health and sexual health, were reported in the persona stories as reasons why young Indigenous people may be reluctant to seek integrated primary care for their mental health needs. Similarly, Robards and colleagues [23] found that marginalised youth often face challenges in knowing how to access services and navigating complex health systems. Current efforts in provincial healthcare fail to recognize and understand how Indigenous people access health care [40]. In the present study, Indigenous youth reported they had ability to navigate online health information, but experienced difficulties to evaluate them. 

The present study provides evidence on the importance of holistic wellness among Indigenous youth. In regard to holistic wellness, many Indigenous people locate the concept of wellness within the Medicine Wheel, containing four balanced aspects of self: mental, physical, spiritual, and emotional [41]. The usability design preferences findings in the current study indicated the importance of the Medicine Wheel and balanced lifestyle in the images and words chosen by Indigenous adolescents. Cultural identity and language are often identified as critical to wellness [41]. This is important, but also not surprising, given that Indigenous persona stories describe a world that is immersed in community and cultural activities where they use digital spaces and community events to ask for health advice from family. In particular, cultural identity was connected to healing and gave a strong sense of pride [41]. 

The present study provides unique findings indicating the relationship with land, sky, and animals. More specifically, design usability preferences were linked to creation stories of Raven, Sacred Tree, and traditional medicines. The interconnectedness between land, sky and animals are culturally embedded in passing on knowledge and intertwined with healing practices [41]. Traditional medicines are important to healing and mental wellness for Indigenous peoples [41]. Cultural practices including smudging, sweat lodges, dances and ceremonies, were important for wellness [41]. The 2017 Aboriginal Peoples Survey found that 71% of First Nations youth reported making an effort to find out more about their history, traditions and culture [16]. In that survey, a majority of Indigenous youth indicated they felt good about their own cultural identity [16].

Regarding social supports, the current study shows the importance of family and social networks. Indigenous young people described the value of informal support from family members and online social networking in the persona stories. This finding is supported in the literature showing community and family were important to Indigenous youth wellness, since they provided cultural connections [41].

Taken together, the findings from the collective storytelling personas and user design preferences provide information about potential barriers to health care access for Indigenous youth who are engaging in eMental health tools. Importantly, this study included a Two-Eyed perspective that bridged together Indigenous collective storytelling not typically utilized in human–computer interaction research. The fact that Indigenous storytelling and oral traditions highlight areas of concern noted by Indigenous youth experiences in integrated primary care access strongly suggests that more work needs to be done to examine the potential of Indigenous storytelling methods in Design Thinking. 

This is the first study to use a Two-Eyed Seeing perspective to utilise Indigenous collective storytelling to empirically examine preferences for digital mental health tools among Indigenous youth. A collective Indigenous storytelling approach used in the present study represents a Two-Eyed Seeing balance between Indigenous oral tradition methods and Western human computer interaction contexts. Stated differently, the Two-Eyed Seeing approach weaved together storytelling and Design Thinking to respectfully engage young Indigenous people to share about their experiences accessing integrated primary care for their mental health needs and preferences for eMental health tools to better understand the wellness needs of Indigenous youth.

### Limitations

First, and most limiting, was the small sample size for the Design Thinking component of this work. The small sample size does not provide a statistically representative understanding of Indigenous adolescents’ lived experiences accessing mental health care. While this might be seen as a small sample size for quantitative studies, the sample size is suitable for human–computer interaction user studies where a typical sample size is 12 [42]. The integration of Indigenous Storytelling and Design Thinking provided participants in an urban setting an opportunity to share a collective story on their experiences accessing integrated primary care for their mental health needs, the meanings they put on digital health tools, and how they express their wellness needs. A limitation of this approach is possible ambiguities, which are inherent in language and storytelling, and can possibly be observed in the present study findings.

## 5. Conclusions

eMental health tools intended to engage Indigenous youth in mental health screening efforts in primary care settings should be designed to include Indigenous knowledges on the importance of mental, emotional, spiritual and physical aspects of a balanced life [43]. A Two-Eyed Seeing approach can inform the development of mental health screening tools and other digital health resources to better support Indigenous adolescents in primary care settings. Yet, few researchers and practitioners have explicitly considered the ways in which a Two-Eyed Seeing approach could be used to integrate Indigenous knowledges into human–computer interaction research to gain insights into Indigenous adolescents’ experiences. Two-Eyed Seeing can provide a link between Indigenous storytelling and design thinking. This method has the potential to provide valuable insight into the underlying mechanisms involved in health seeking behaviours and engagement with digital health apps for Indigenous adolescents.

## Figures and Tables

**Table 1:** Demographic Information for Design Circle Participants.

Demographic Characteristic	Design Circle
	1 and 2 (n = 8)
Gender	
Young Man	3
Young Woman	4
Two Spirit	1
Indigenous	
First Nations Status	6
First Nations Non-Status	2
Highest Education	
High School Graduate	4
Further/vocational education	1
Higher education	2
Prefer not to say	1
Sexual Orientation	
Heterosexual	6
LGTBQIA2S+	1
Prefer not to say	1
Adults You Live with Most *	
Mother or Stepmother	1
Father or Stepfather	2
Grandparents	0
Sibling	2
Other Family	1
Foster Parents	0
Do not live with caregiver	6
Length of Time Living in Urban Area	
6 months to 1 year	2
1 to 2 years	0
2 to 3 years	1
3 to 5 years	0
5 to 10 years	1
More than 10 years	4
Do You Have a Family Physician?	
Yes	4
No	3
Prefer to not say	1

* Participants were able to select more than one response for adults they live with.

**Table 2:** eHealth Literacy Scale (eHEALS) mean items scores for Design Circle Participants.

Item	Mean (SD)
I know how to find helpful health resources on the Internet	3.88 (0.99)
I know how to use the Internet to answer my health questions	3.88 (0.64)
I know what health resources are available on the Internet	3.63 (0.92)
I know where to find helpful health resources on the Internet	3.88 (0.35)
I know how to use the health information I find on the Internet to help me	3.75 (0.46)
I have the skills I need to evaluate the health resources I find on the Internet	3.50 (0.54)
I can tell high quality from low quality health resources on the Internet	3.38 (0.74)
I feel confident in using information from the Internet to make health decisions	2.88 (1.13)

**Table 3:** Trustworthiness of Online Health Information.

Item	Design Circle
	n (%)
It came up at the beginning of the search results	2 (25)
By recognizing the group or person who created the information	4 (50)
Person or group responsible for the website is an expert (for example, a doctor or nurse)	2 (25)
The website belongs to a health organization, hospital, or government	5 (63)
It does not have any advertising or doesn’t try to sell me something	1 (13)
By looking at the last time that the information was updated	2 (25)
The website belongs to people that I can relate to	0
A friend recommended the webpage	1 (13)
A healthcare professional (for example a doctor or nurse) recommended the website	3 (38)
The information is easy to read and understand	2 (25)
The website has contact information	3 (38)
I don’t want to answer this question	1 (13)
None of the above	2 (25)

**Table 4:** Design Circle Schedule.

Topic Areas	What Youth Will Do	Time
Session Plan the First Design Circle		
Topic Area 1: Design Circle Overview and introductions	Participant Consent	20 min
	Research Team Intro and How the Design Circle Will Work	10 min
	Introduction Ice-Breaker Activity	15 min
Topic Area 2: Participatory Design Activity	Introduction to mobile app	5 min
	Create Persona Stories—create random groups of 2 to 3 participants	15 min
	Persona prototyping	20 min
Break	Lunch Provided	30 min
Topic Area 2 Continued	Combine designs—large Circle group discussion	20 min
Topic Area 3: Card Sorting Activity	Sorting Usability Preferences—use the same groups of 2 to 3 participants from participatory design activity	30 min
Topic Area 4: Wrap up and Questionnaire	Closing the Circle (distribute honorarium)	5 min
	Handout the two-page demographic questionnaire	5 to 10 min
Session Plan for the Second Design Circle		
Topic Area 1: Design Circle Overview and introductions	Welcoming Remarks and Review How the Design Circle Will Work	15 min
	Introduction Ice-Breaker Activity	15 to 20 min
Topic Area 2: Participatory Design Activity	Further design work to Mobile app—create new random groups of 2 to 3 participants	45 min
Break	Lunch Provided	30 min
Topic Area 3: Card Sorting Activity with Aboriginal Perspectives on Health and Wellness	Identify Indigenization and Design Goals using Aboriginal Health Perspective	5 min
	YouTube Clip Showing Ideas: - News Story on Aboriginal People’s Experience in Canadian Healthcare (https://www.youtube.com/watch?v=IXr-Cfj3EPM, accessed on 17 August 2016) - Smoke Signals Movie Clip (https://www.youtube.com/watch?v=uwcJaUaVfR0, accessed on 17 August 2016) - Leonardo DiCaprio’s Golden Globe Tribute to Indigenous People (https://www.youtube.com/watch?v=ncgFQAISaGo, accessed on 17 August 2016)	5 to 10 min
	YouTube Videos Debrief: Pair and Share Activity: Youth asked to partner with the participant sitting next to them in the Circle and most comfortable with.	10 min
	Discussion Question: What specific things in the videos and First Nations holistic health perspective did you personally find the most interesting or useful?	40 min
Topic Area 4: Wrap up	Closing remarks: Distribute honorarium	5 min

## Data Availability

Data are available on request from researchers.

## Supplementary Materials

The following supporting information can be downloaded at: https://www.mdpi.com/article/10.3390/ijerph192214972/s1, Table S1: Access, Device and Internet Use.
