# Supplementary material for: Application of Two-Eyed Seeing in Adolescent Mental Health to Bridge Design Thinking and Indigenous Collective Storytelling
Source: Int J Environ Res Public Health. 2022 Nov 14;19(22):14972. doi: 10.3390/ijerph192214972 (PMC9690396; doi:10.3390/ijerph192214972)
Supplement: Supplementary file 1 [file ijerph-19-14972-s001.zip › ijerph-1924580-supplementary.pdf]

Supplemental Table S1. Access, Device and Internet Use

	Design Circle 1 and 2 (n = 8)
Type of Device to Access Internet*	
Cell or mobile phone	3
Desktop or laptop computer	1
Tablet or iPad	0
MP3 player or iPod	0
Game console (e.g. Playstation, XBox, Nintendo)	0
2 devices selected	4
Days per Week Online	
Seven days a week	6
Four days a week	1
Missing	1
Importance to have Internet Access	
Extremely important	1
Very Important	1
Important	3
Somewhat important	3
Has own Device to Access Internet	
Yes	6
No	1
Number of hours on Internet per day Weekdays	
Mean 5.9, Standard Deviation 4.9, Median 4.5, Minimum 1, Maximum 14	
Number of hours on Internet per day Weekdays	
Mean 5.6, Standard Deviation 5.3, Median 4, Minimum 1, Maximum 16	

* Participants were able to select more than one response.
